# The burden of trisomy 21 disrupts the proteostasis network in Down syndrome

**DOI:** 10.1371/journal.pone.0176307

**Published:** 2017-04-21

**Authors:** Stefanos Aivazidis, Christina M. Coughlan, Abhishek K. Rauniyar, Hua Jiang, L. Alexander Liggett, Kenneth N. Maclean, James R. Roede

**Affiliations:** 1 Department of Pharmaceutical Sciences, Skaggs School of Pharmacy and Pharmaceutical Sciences, University of Colorado, Aurora, CO, United States of America; 2 Department of Neurology, University of Colorado School of Medicine, Aurora, CO, United States of America; 3 The Linda Crnic Institute for Down Syndrome, University of Colorado, Aurora, CO, United States of America; 4 Department of Pediatrics, University of Colorado School of Medicine, Aurora, CO, United States of America; University of Pittsburgh, UNITED STATES

## Abstract

Down syndrome (DS) is a genetic disorder caused by trisomy of chromosome 21. Abnormalities in chromosome number have the potential to lead to disruption of the proteostasis network (PN) and accumulation of misfolded proteins. DS individuals suffer from several comorbidities, and we hypothesized that disruption of proteostasis could contribute to the observed pathology and decreased cell viability in DS. Our results confirm the presence of a disrupted PN in DS, as several of its elements, including the unfolded protein response, chaperone system, and proteasomal degradation exhibited significant alterations compared to euploid controls in both cell and mouse models. Additionally, when cell models were treated with compounds that promote disrupted proteostasis, we observed diminished levels of cell viability in DS compared to controls. Collectively our findings provide a cellular-level characterization of PN dysfunction in DS and an improved understanding of the potential pathogenic mechanisms contributing to disrupted cellular physiology in DS. Lastly, this study highlights the future potential of designing therapeutic strategies that mitigate protein quality control dysfunction.

## Introduction

Down syndrome (DS) is a genetic disorder resulting from the triplication (whole or part) of chromosome 21 (Hsa21)[1]. While DS is a form of aneuploidy in humans, it is the only trisomy that does not result in embryonic or early life lethality [2]. Chromosome missegregation has been shown to lead to increased mRNA production and excessive protein formation; thus, linking aneuploidy with a disruption of the proteostasis network and the production of proteotoxic stress [3, 4]. Due to trisomy 21, the DS population is characterized by a variable phenotype with several comorbidities. These comorbidities include seizures [5, 6], leukemia [7], vision problems [8], thyroid dysfunction [9], diabetes [10] and dementia, specifically early onset Alzheimer’s disease (AD) [11]. Lastly, errors in protein homeostasis are proposed as candidate mechanisms related to the pathology of the aforementioned comorbidities [12–20].

**Fig 1:**
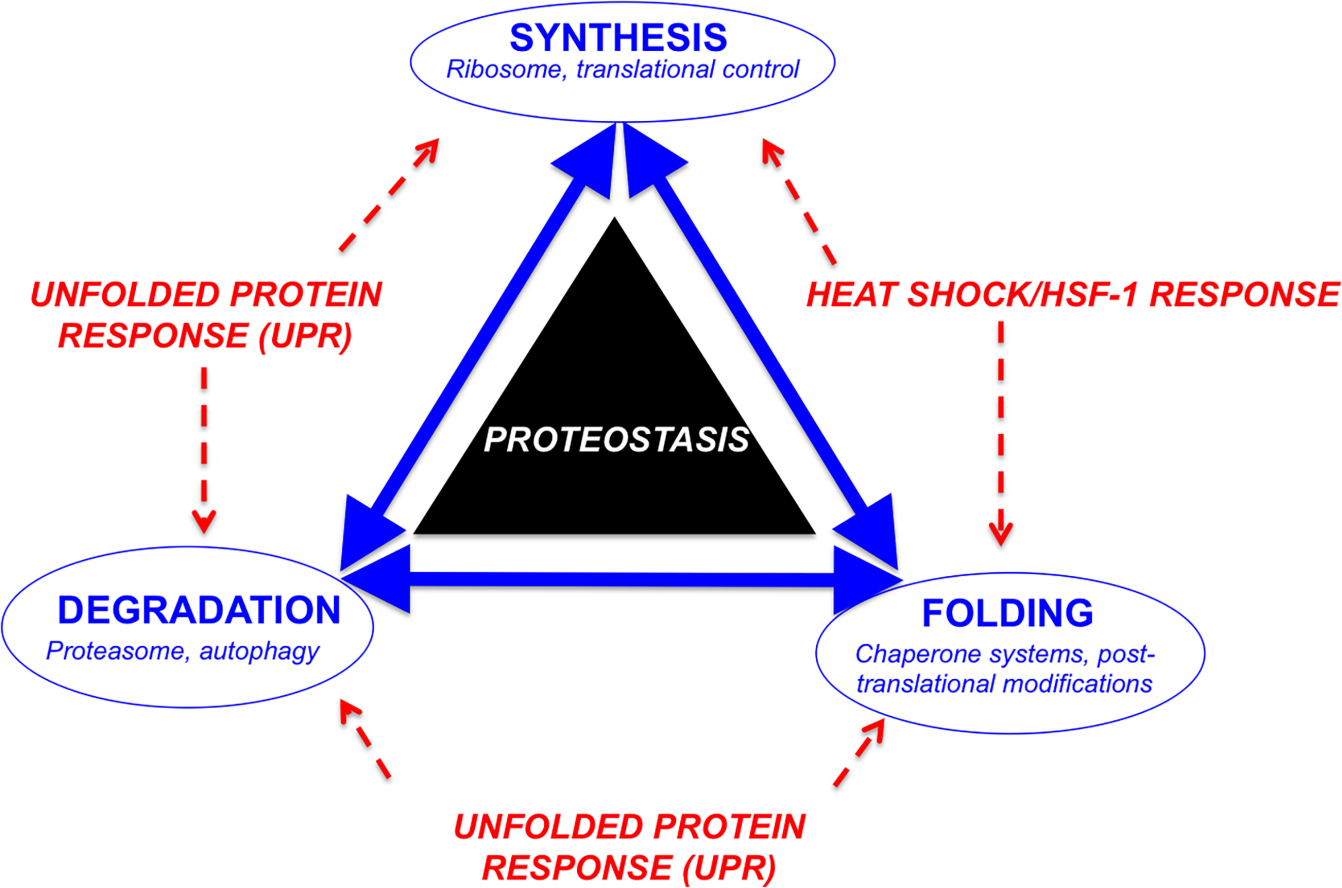
A simplified schematic illustrating the three major functions of the proteostasis network (Blue), and the key modulators (Red), UPR and HSF-1.

Protein homeostasis, or proteostasis, refers to the correct function and balanced abundance of the cellular proteome. The proteostasis network (PN) is the system responsible for maintaining the stability and integrity of the proteome. Synthesis of proteins, as well as proper protein folding, repair/disaggregation, and clearance/degradation are major components of the PN (Fig 1). The PN includes the ribosome, molecular chaperones, and degradation machinery involved in the proteasome and autophagy [21]. In addition, there are several PN modulators, like the unfolded protein response (UPR) and the transcription factor heat shock factor-1 (HSF-1), that modify the PN, and disruption or alteration of these modulators can lead to the distortion of the PN architecture [22, 23]. Finally, malfunction of the PN has been proposed as an etiologic factor for the promotion of certain diseases, as PN imbalance leads to misfolded protein accumulation and proteotoxic stress [24].

Since alterations in the PN occur in various pathological conditions, e.g. diabetes, AD, vision-related defects, thyroid dysfunction, and epilepsy, we hypothesized that, due to the presence of these comorbidities in the DS population, cells from DS individuals will display a dysfunctional PN. Also, the burden of trisomy, mediated by gene dosage effects [25], has the potential to disturb proteostasis by overwhelming the cellular translational quality control mechanisms, that over time would act as a mechanism to promote disease pathology and decrease cell viability. Consistent with our hypothesis, we observed several components of the protein quality control machinery in DS cell models to be dysfunctional compared to euploid controls. Furthermore, treatment with stressors that disrupt the PN resulted in decreased cell viability in DS cells compared to controls. Our basic findings from this characterization suggest that therapeutic strategies, designed to alleviate dysfunction of the PN, will promote clearance of misfolded proteins, potentially resulting in significant therapeutic outcomes in the DS population.

## Materials and methods

### Reagents and antibodies

Maneb (Product number 45554) and tunicamycin (Product number T7765) were purchased from Sigma-Aldrich. 4μ8c (Product number 412512) was purchased from EMD Millipore. All other reagents were purchased from Fisher-Scientific. The following primary antibodies were purchased from Cell Signaling Technology: XBP1s (D2C1F) (12782), ubiquitin (3933), phospho-eIF2α (Ser51) (9721), eIF2α (9722), IRE1α (3294), HSP40 (4871), HSP70 (4872), HSP90 (4877). The following primary antibodies were purchased from Abcam: ATF6 (ab122897), and XBP1 (ab37152). The primary antibody against phospho-IRE1α (Ser724) was purchased from Novus Biologicals. The primary antibody against HSP27 was purchased by Enzo Life Sciences (G3.1). The primary antibody against β-actin (A5441) was purchased from Sigma-Aldrich.

### Cell culture

Lymphoblastoid cell lines (LCL), immortalized using the Epstein-Barr virus [26], were obtained from the Intellectual and Developmental Disabilities Research Center (IDDRC) Nexus (Colorado Multiple Institutional Review Board (COMIRB) #08–1276) in the Department of Pediatrics at the University of Colorado Anschutz Medical Campus. LCLs from three DS patients were used (female, 18.4 yrs old; male, 18.3 yrs old; female, 7.9 yrs old) and three non-DS, age-matched controls were used as euploid controls (CTL) (female, 17.9 yrs old; male, 16.4 yrs old; female, 7.5 yrs old). The LCLs were cultured in RPMI 1640 medium containing L-glutamine, 15% FBS, and 1% antibiotic/antimycotic solution. For mechanistic studies, we utilized human fibroblasts derived from the foreskin of DS and euploid patients. Detroit 551 (CCL-110), a disomic cell line used as euploid control (CTL) in our experiments, and Detroit 539 (CCL-84), a trisomic cell line derived from a DS patient were purchased from ATCC. The CCL-110 cell line was cultured in EMEM medium supplemented with 10% FBS, 1% antibiotic/antimycotic solution. The CCL-84 cell line was cultured in EMEM medium with 10% FBS, 1% antibiotic/antimycotic and 0.1% lactalbumin.

### Animal tissues

Wild type (WT), Dp(16)1Yey/+ (DP16), Dp(17)1Yey/+ (DP17) (n = 3/strain) were obtained from the Linda Crnic Institute for Down Syndrome. All animal functions were governed by protocols approved by the University of Colorado Institutional Animal Care and Use Committee. The animals were all males and aged approximately 10–12 weeks. Upon receipt of the animals from the animal core, they were euthanized via CO_2_ inhalation and cervical dislocation. Tissues were harvested immediately and flash frozen in liquid nitrogen. Approximately one complete hemisphere of the brain was homogenized in RIPA buffer containing Halt Protease and Phosphatase Inhibitor cocktail (Fisher Scientific). Samples were aliquoted and stored at -80°C until use. Protein concentration of each sample was determined using a BCA assay (Fisher Scientific).

### Quantitative real-time PCR (qPCR)

RNA was extracted from DS and euploid control LCLs using an RNeasy mini kit (Qiagen). RNA (2 μg) was reverse transcribed to cDNA using iScript Advanced cDNA Synthesis Kit (Bio-Rad). For qPCR, approximately 200 ng of cDNA was utilized. PrimePCR Sybr Green assays were purchased from Bio-Rad for the following genes: GADD153, ATF6, GADD34, CNE, HSPA5, HSP90B1, XBP1, and PDIA3. HPRT1 was utilized as a loading control. Samples were analyzed using a CFX Connect 96-well real-time PCR detection system (Bio-Rad) as per manufacturer’s protocols.

### Detection of reactive oxygen species

To determine superoxide production, untreated LCLs were stained with reagents contained in the Muse Oxidative Stress kit (Cat # MCH100111, Millipore) and fluorescence was detected using a Muse Cell Analyzer (Millipore) as per manufacturer’s protocol. Hydrogen peroxide production was determined in untreated LCLs utilizing a Total Reactive Oxygen Species (ROS) assay kit (Cat # 88-5930-74) from eBioscience as per manufacturer’s instructions.

### Isolation of nuclear and cytosolic fractions

To isolate nuclear and cytosolic fractions from CTL and DS fibroblasts, the NE-PER nuclear extraction kit (Thermo Scientific) was used as per manufacturer’s protocol. Briefly, CCL-110 and CCL-84 cells were grown on 100mm culture plates and allowed to achieve 70–90% confluence. The cells were then dissociated from the plate using 0.25% trypsin with EDTA. The cells were then pelleted by centrifugation and washed twice with phosphate buffered saline. The washed cells were pelleted again and the nuclear extraction procedure was commenced following the manufacturer’s protocol.

### Western blotting

20–40 μg of each protein lysate was separated via SDS-PAGE utilizing a 10% polyacrylamide gel. Proteins were transferred to nitrocellulose membrane using the Bio-Rad Trans-Blot semi-dry transfer apparatus. Blocking was performed for 20 min using 5% nonfat dried milk in TBS-0.1% Tween (TBS-T). Primary antibodies were diluted 1:1000 in TBS-T containing 10% Super Block T20 (Thermo Scientific) and incubated with the blot overnight at 4°C. After three washes for 10 min in TBS-T, the blot was incubated with a horseradish peroxidase (HRP) -conjugated secondary antibody at 1:5000, diluted in TBS-T containing 10% Super Block T20. Clarity Western ECL Substrate (Bio-Rad) was used to detect the HRP of the secondary antibody. Imaging was performed on the ChemiDoc MP imaging system and Image Lab software (Bio-Rad). These experiments were conducted in at least three independent replications, and the image is a representative sample.

### Proteasomal activity assessment

Proteasomal activity was assessed as previously described [27] by measuring the fluorescence of three different fluorogenic peptides that serve as proteasomal enzyme substrates (chymotrypsin-like activity (UBPBio: Cat. # G1100,G1101), caspase-like activity (Calbiochem: Catalog number 539141) and trypsin-like cleavage activity (Peptide Institute, Inc.: Catalog Code Number: 3140-v). Briefly, cells were harvested in assay buffer containing 50mM Tris-HCl (pH 7.5), 250mM sucrose, 1mM dithiothreitol, 5mM MgCl_2_, 2mM ATP, 0.5mM EDTA, and 0.025% (w/v) digitonin, and centrifuged at 10,000xg for 14min. Supernatants were assayed from protein concentration using a BCA assay. Proteasome activity was determined by incubating 20μg protein with 100μM fluorogenic peptide in assay buffer (total volume—200μl per reaction) for 30min at 37°C. The reaction was halted by the addition of 200μl of ice-cold ethanol (100%) and the reactions were centrifuged for 4min at 10,000xg. Supernatant was transferred to a black, 96-well plate and fluorescence (Ex. 380nm, Em. 460nm) was measured using a fluorescent plate reader (Molecular Devices). All samples were analyzed using at least 3 analytical replicates and the assay was conducted at least twice for reproducibility.

### Cell viability assay

For the evaluation of cell viability, CTL and DS fibroblasts were plated in a 96-well plate (10,000 cells per well) and allowed to recover and adhere overnight. Cells were treated with increasing concentrations of maneb (MB) or tunicamycin (Tm) for 24h. After the 24h treatment, a WST-1 colorimetric assay (Roche, Product number: 05015944001) was used to assess cell viability as per the manufacturer’s protocol. A SpectraMax 190 microplate reader (Molecular devices, Sunnyvale CA, USA) was used to read the absorbance at 450nm.

### Immunocytochemistry

CTL and DS fibroblasts were seeded onto coverslips and allowed to adhere and recover for 16h. The coverslips were then placed into individual wells of a 6-well plate for treatment. Cells were then treated for 24h with vehicle (DMSO) or proteasome inhibitor (MG132, 5μM). Upon completion of the treatment, cells were fixed with 4% (v/v) paraformaldehyde in PBS and antigens were retrieved using 0.1% (v/v) triton-X 100 in PBS. Cells were then blocked for 30 minutes at room temperature using a 1:1 mixture of TBS-T and complete culture medium (EMEM, 10% FBS, 1% penn/strep). Slides were then incubated with a primary antibody against ubiquitin (Cell Signaling Technology, 3933) overnight at 4°C. The coverslips were then gently washed 3 times using TBS-T followed by incubation with a TRITC-labeled secondary antibody and DAPI (1μg/ml) for 1h in the dark at room temperature. Coverslips were washed 3 times in TBS-T, dipped in distilled water then mounted on slides using SuperMount (BioGenex) and allowed to dry. Cells were imaged using a Nikon TE2000 microscope with a Nikon C1 confocal imaging system. Each coverslip was imaged ten times (ten different fields) and this experiment was conducted in three independent trials for a total of 30 images/fields per treatment and genotype. Analyses of the confocal images were performed as per Orlicky et al [28]. Briefly, TIFF images were captured in RGD and the ICC signal of ubiquitin was quantified in these images using the 3I Slidebook program (3I, Denver, Colorado). Similarly, the signal from the DNA bound DAPI dye was also quantified. Data is presented as the ICC signal normalized to the quantity of DNA present.

### Statistics

Data is represented as the mean ± standard error of the mean (SEM). All experiments were performed in duplicate or triplicate. Data were analyzed and graphs were plotted using GraphPad Prism 6. Statistical significance was determined using unpaired t-test and a P-value of <0.05 was deemed to be significant (* P<0.05; ** P<0.01; *** P<0.001; **** P<0.0001).

## Results

### Lymphocytes from DS patients elevated basal reactive oxygen species production and increased expression of ER stress-related genes

**Fig 2:**
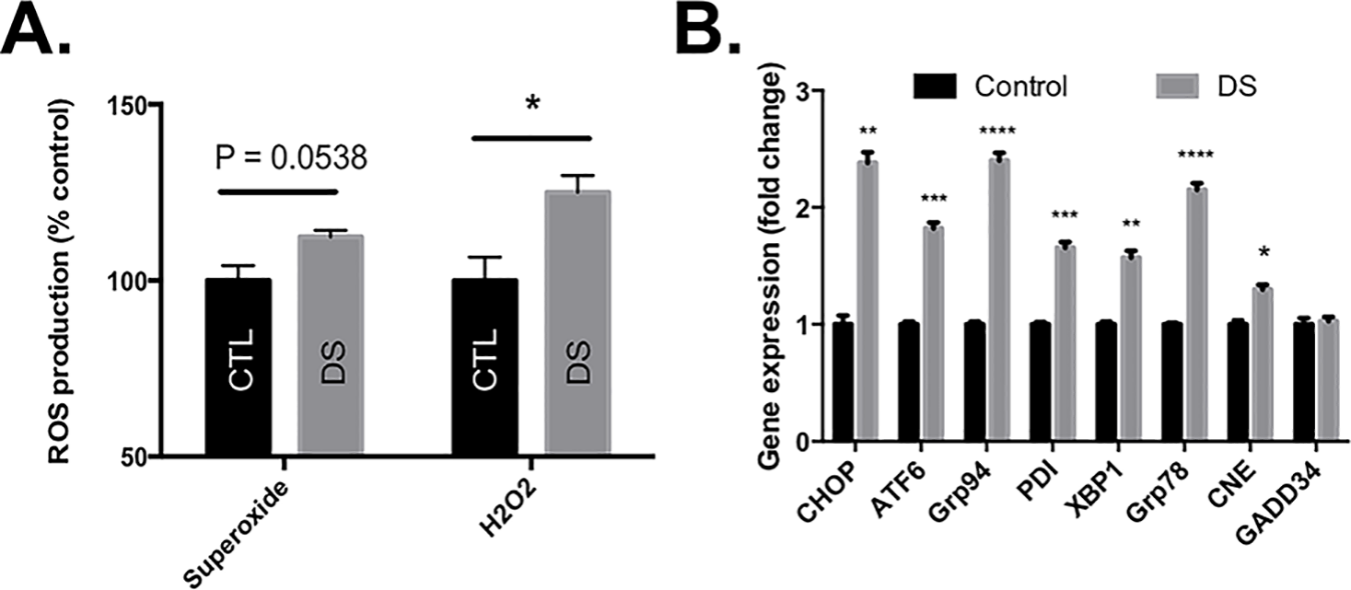
**LCLs from DS patients enhanced basal levels of ROS production (A) and expression of ER stress genes (B).** Unstimulated DS and CTL LCLs were evaluated for basal superoxide and hydrogen peroxide production using flow cytometry. These unstimulated cells were also assessed for ER stress gene expression using qRT-PCR. N = 3; * P<0.05; ** P<0.01; *** P<0.001; **** P<0.0001.

DS is a “whole body” condition that involves multiple comorbidities that impact nearly all body systems [29–32]; therefore, we chose to employ two different cell culture models of cells that can be obtained from DS individuals in a relatively non-invasive manner, lymphoblastoid cells (LCL) and skin fibroblasts. The presence of basal oxidative stress is a common observation in DS [33–35], and this was confirmed in our LCL model by measuring hydrogen peroxide and superoxide production in unstimulated cells (Fig 2A). DS cells displayed a significant increase in hydrogen peroxide production, while no significant change in superoxide was observed. The enhanced hydrogen peroxide production in the DS cells is potentially attributable to the elevated levels of SOD1 protein present in these cells [36, 37], which converts superoxide to hydrogen peroxide. Additionally, reactive oxygen species can damage cellular macromolecules, e.g. proteins, and it is known that oxidative damage to proteins can result in misfolding and altered proteasomal degradation; therefore, cells from DS individuals may display alterations to the PN. To examine this possibility we measured the mRNA expression levels of several UPR-related genes in LCLs derived from DS individuals (Fig 2B) versus euploid controls. Consistent with our hypothesis, our data show a modest, but significant up-regulation in the expression of UPR-related genes (CHOP, ATF6, XBP1, PDI, GRP78, GRP94, CNE). We also investigated the ability of DS cells to activate UPR using the thiol-reactive fungicide maneb as an UPR inducer. These results demonstrated that DS cells, in addition to having elevated basal markers of UPR, can adequately induce Grp78 and XBP1s abundance and eIF2α phosphorylation (S1 Fig). Collectively, these data indicate that cells from DS patients exhibit constitutive induction of the UPR, increased basal levels of oxidative stress, and are capable of stress-induced UPR activation.

### XBP1s protein abundance is elevated in DS cells and DP16 mice

**Fig 3:**
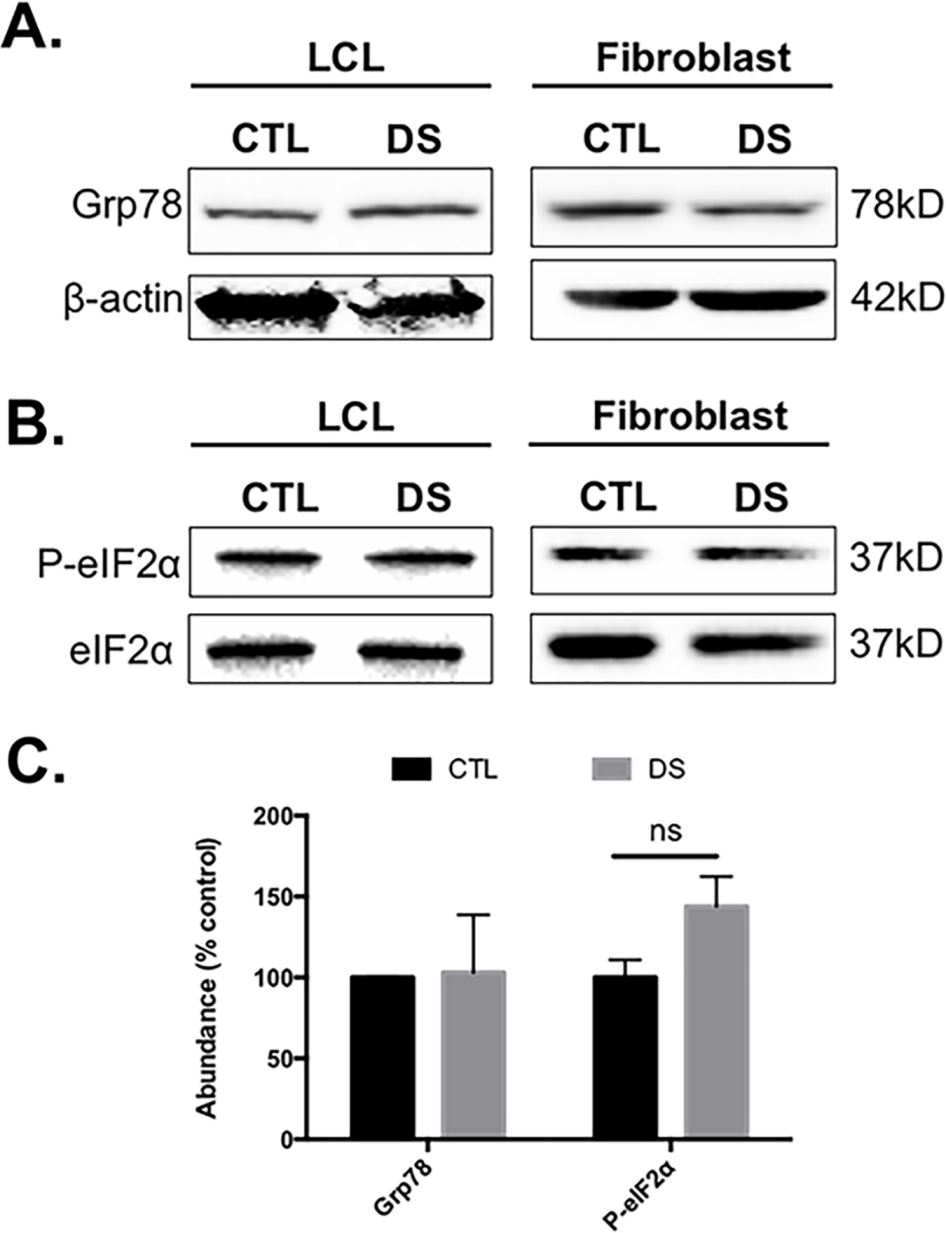
Grp78 and the PERK-eIF2α pathway are not basally up-regulated in DS LCLs or fibroblasts. Western blot analyses in both LCLs and fibroblasts from DS and euploid controls did not show a difference in either basal Grp78 abundance (A) or abundance and phosphorylation status of eIF2α (B).

**Fig 4:**
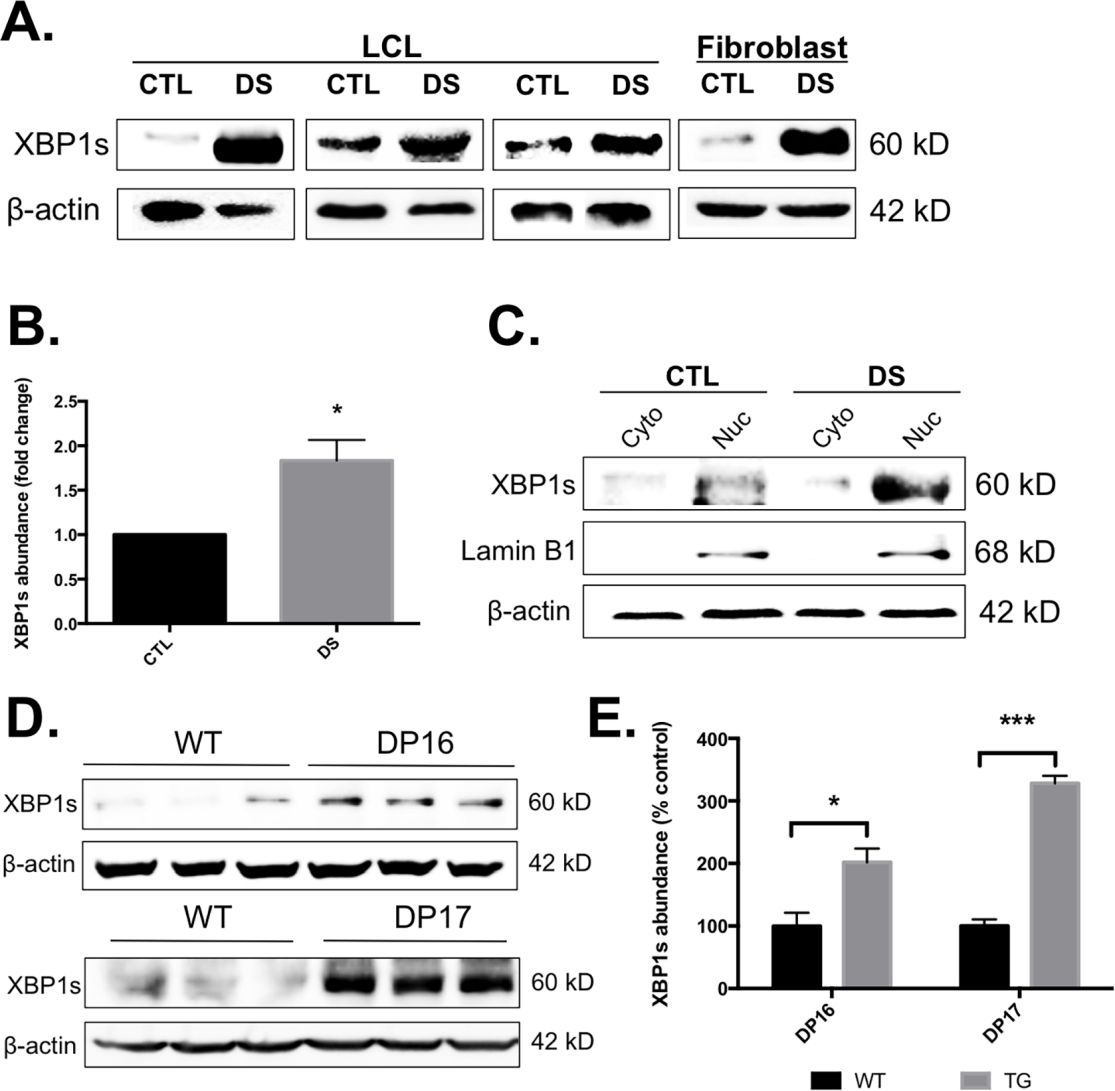
LCLs and fibroblasts from DS patients and mouse models of DS display increased abundance of XBP1s and XBP1s was found localized to the nucleus. Unstimulated LCLs and fibroblasts from DS individuals and age-matched controls were examined for XBP1s abundance using Western blot (A-C). Both DP16 and DP17 (D) mice display significant increase in XBP1s abundance in the brain (E). Western blots were conducted in triplicate and the images are representative of these replications. WT, wild type; TG, transgenic (DP16 or DP17); n = 3, * P<0.05.

The gene expression study described above indicated that DS cells exhibit enhanced basal levels of UPR. Because of this observation, we sought to investigate any DS-mediated difference in the abundance of various UPR proteins. We evaluated all three major arms of UPR using LCLs from three different DS individuals and age-matched controls, as well as commercially available DS (CCL-84) and euploid control (CCL-110) skin fibroblasts. These studies revealed no significant differences in the phosphorylation state or abundance of eIF2α, as well as no significant difference in basal GRP78 abundance (Fig 3A and 3B). It should be noted that GRP78 abundance was increased in DS LCLs, but not fibroblasts, indicating a possible cell type-specific difference. However, we did observe an increased abundance of the activated form of X-box binding protein 1 **(**XBP1s) in the DS cells compared to euploid controls (Fig 4A and 4B).

Since XBP1s is a transcription factor, it’s increased splicing would imply increased nuclear localization in DS versus euploid controls. When examined, we did observe a greater abundance of XBP1s in the DS nucleus compared to euploid controls (Fig 4C). In an effort to examine the significance of our XBP1s expression data, we investigated the abundance of XBP1s in the brains of WT, and two segmental trisomy models of DS, DP16 and DP17 mice. Due to the fact that the orthologous genes located on Hsa21 are located on mouse chromosome 10, 16 and 17, segmental trisomy models have been created that are segmental trisomy for the genes located on mouse chromosome (Mmu) 10 (41 Hsa21 orthologs), Mmu16 (115 Hsa21 orthologs), and Mmu17 (19 Hsa21 orthologs) [38]. As described in the Materials and Methods section, one complete hemisphere of the brain was homogenized and analyzed via Western blotting; therefore, these results correspond to whole brain and not a specific brain region. The total protein abundance of XBP1s in both DS mouse models was significantly increased (Fig 4D and 4E) and is consistent with the data obtained from human DS cell lines.

### IRE1α hyperphosphorylation is not responsible for the elevated abundance of XBP1s

**Fig 5:**
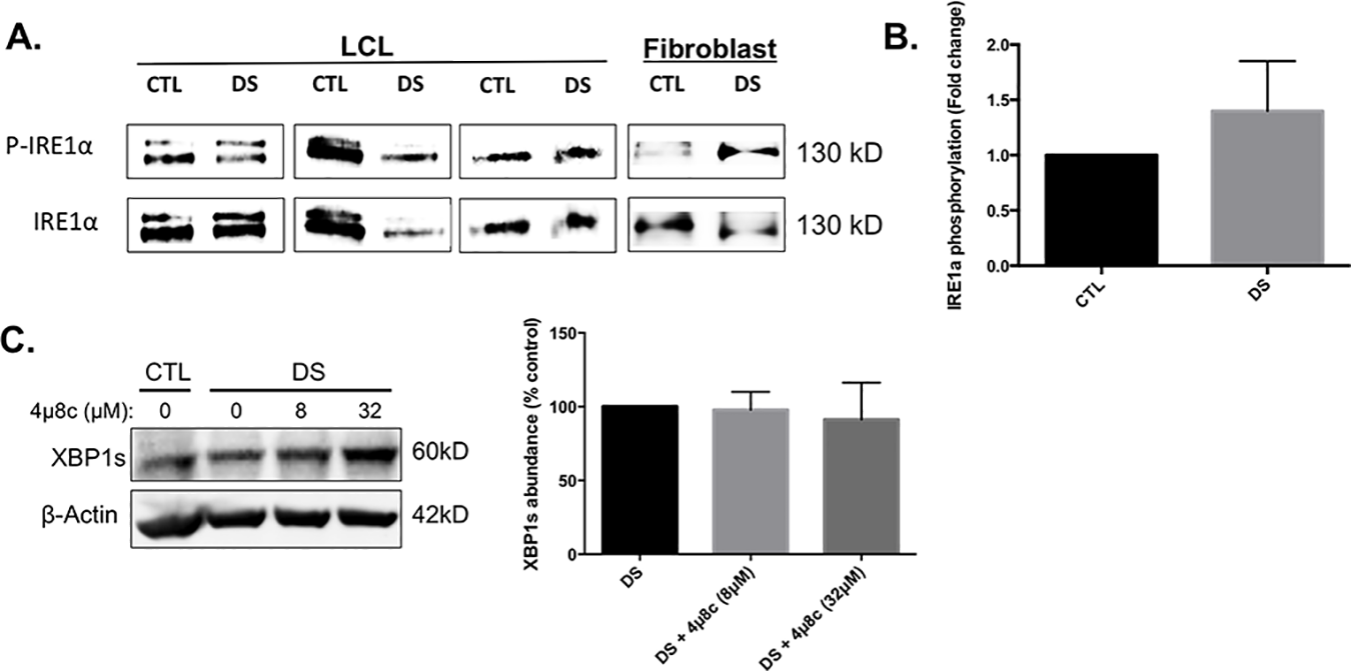
Phosphorylation of IRE1α does not differ greatly between cells from DS and euploid controls. Three pairs of DS and CTL LCL, as well as one pair of DS and CTL fibroblasts, were analyzed for basal levels of IRE1α phosphorylation (A). These data show that LCLs did not display a clear DS-mediated phenotype, while the fibroblasts showed a DS-mediated increase in phosphorylation (B). Inhibition of the IRE1α endonuclease domain with 4μ8c did not significantly decrease XBP1s abundance in DS fibroblasts (C). Western blots were conducted in triplicate and the images are representative of these replications.

Phosphorylation of IRE1α activates the cytoplasmic endonuclease domain of IRE1α, leading to XBP1u mRNA cleavage to XBP1s, resulting in the expression of an active transcription factor [39]. Therefore, in an effort to understand the mechanism by which DS cells up-regulate the abundance of XBP1s protein we evaluated the phosphorylation state of IRE1α. Data presented in Fig 5A and 5B show that DS LCLs do not display increased IRE1α phosphorylation compared to controls; however, DS fibroblasts did display increased IRE1α phosphorylation compared to controls. To further investigate the role of IRE1α in this study, we treated CTL and DS fibroblasts with the small molecule inhibitor of the IRE1α endonuclease, 4μ8c, for 24h and performed Western blots to determine the abundance of XBP1s. Fig 5C shows that this inhibitor did not alter the abundance of XBP1s in DS cells, indicating a possible alternative route for XBP1 mRNA splicing or an enhanced half-life of this protein in DS cells possibly due to proteasomal dysfunction or an unknown posttranslational mechanism. It should be noted that Rutkowski and colleagues have recently shown that XBP1 mRNA splicing can be negatively impacted in a condition of chronic ER stress in the liver [40]; however, regulated IRE1α dependent decay (RIDD) was still active in these hepatocytes, potentially indicating alternate avenues for RNA splicing and UPR signaling. Due to these variable and potentially cell type specific responses, we hypothesized that additional mechanisms must be responsible for the observed increase in XBP1s abundance.

### Increased activation to ATF6 may be responsible for increased expression of XBP1s

**Fig 6:**
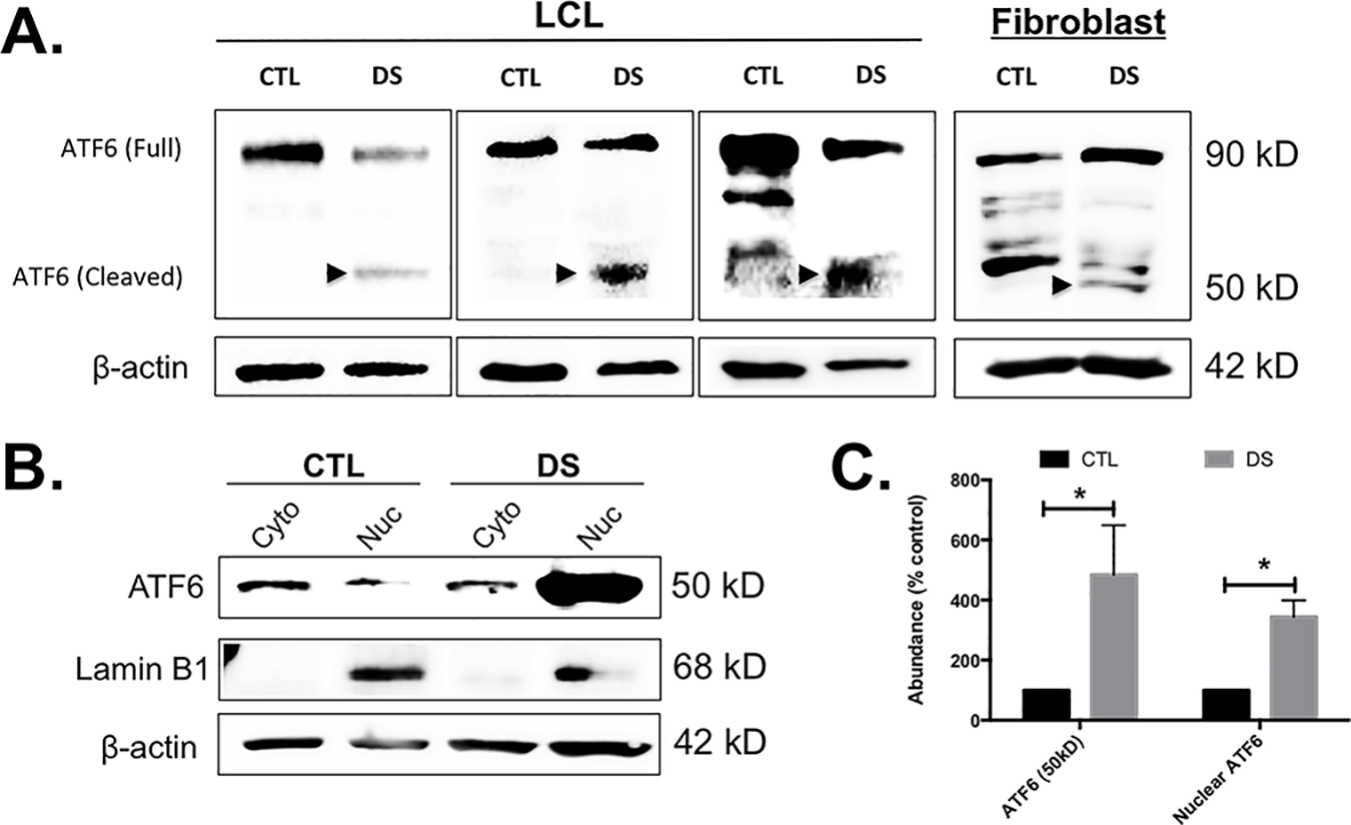
**LCLs and fibroblasts from DS patients show increased ATF6 cleavage (A) and increased nuclear localization (B) compared to euploid controls.** Both DS LCLs and fibroblasts displayed increased ATF6 cleavage, as evidenced by the presence of a band located at approximately 50 kD (arrow). Significantly increased ATF6 cleavage and nuclear localization was observed in the DS cells compared to euploid controls (C). Western blots were conducted in triplicate and the images are representative of these replications.

The XBP1 gene is reported to be a downstream target of ATF6 [41] and this transcription factor could conceivably be a mechanism for XBP1 gene induction observed here (Fig 2 and Fig 4). Similar to XBP1s, upon release of ATF6 from the ER and cleavage in the Golgi, the protein is translocated to the nucleus. Western blot analyses of patient-derived LCLs and fibroblasts showed that DS cells possess a greater amount of cleaved/activated (50kD fragment) ATF6 protein (Fig 6A), a robust increase in ATF6 nuclear localization in unstimulated DS cells (Fig 6B), and these alterations were found to be statistically significant when compared to euploid controls (Fig 6C). These data suggest that DS-mediated ATF6 activation may be responsible for the increase in XBP1s protein in DS cells.

### DS fibroblasts display impaired induction of chaperones in response to moderate heat stress

**Fig 7:**
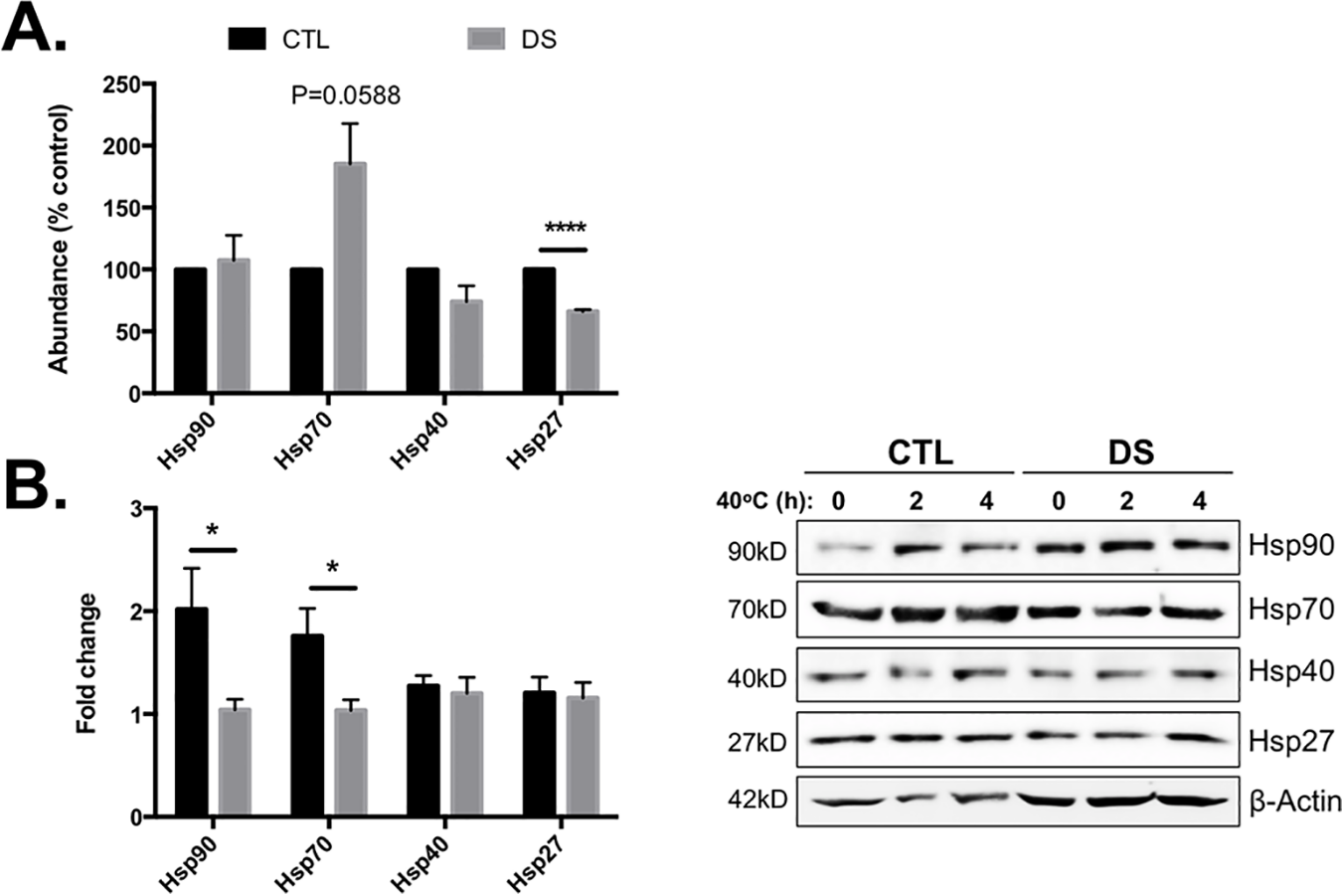
Impaired heat shock response in DS fibroblasts. Basal levels of most heat shock proteins (Hsp) investigated were not significantly different controls; however, Hsp27 was significantly decreased in DS cells (A). Stimulation of these cells for 2h with 40°C heat stress did not result in a significant increase in Hsp90 or 70 (B). These results indicate an abnormal HSF-1 response in DS fibroblasts. Graphs represent results of Western blotting experiments that were conducted in triplicate. Data is represented as the mean ± SEM (*P<0.05, ****P<0.0001).

In addition to modification by induction of UPR, the PN also includes greater than 300 chaperone genes that make up the chaperome [42]. These genes are charged with assisting in protein folding and mediating solubility and function of much of the proteome [43]. Due to the observation of basal activation of UPR in DS cells, we next investigated the basal levels of a small sample of chaperone proteins and the response of DS and CTL fibroblasts to moderate heat stress (40°C). Fig 7A shows that CTL and DS cells possess similar abundance of Hsp90, Hsp70, and Hsp40; however, the basal abundance of Hsp27 was found to be significantly decreased in DS compared to CTL. Upon stimulation of these CTL and DS cells with 40°C heat stress for 2 hours, it was discovered that cells from DS individuals do not respond in a manner similar to that of the CTL (Fig 7B). Thus, in addition to elevated UPR, cells from DS individuals have a severely blunted or absent response to moderate heat stress and do not induce chaperone proteins in a manner similar to CTL.

### DS fibroblasts exhibit increased protein ubiquitination and disrupted proteasome activity

**Fig 8:**
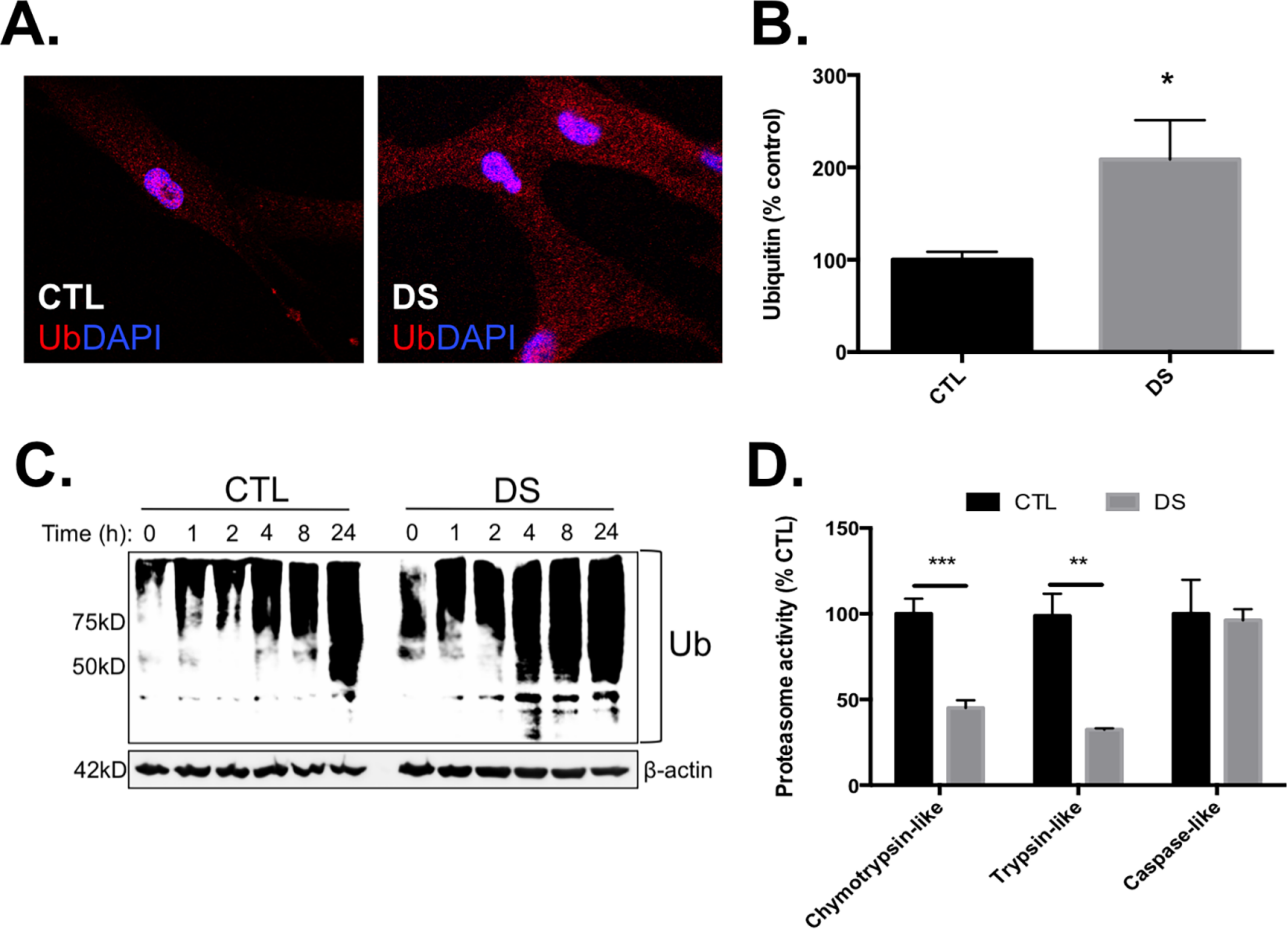
DS cells display an increased abundance of ubiquitinated proteins and impaired proteasomal function. Untreated fibroblasts from DS and CTL were stained for ubiquitinated protein and visualized using fluorescent microscopy (A). The amount of basal ubiquitin staining was found to be significantly increased in DS fibroblasts compared to CTL (B), indicating increased load of misfolded proteins in DS cells. Western blot analyses after treatment with the proteasomal inhibitor, MG132 (5μM), show a more rapid increase in polyubiquitinated proteins (C) in DS compared to CTL. Further investigation of proteasomal activity demonstrated that in DS fibroblasts there are significant decreases in both chymotrypsin-like and trypsin-like activity of the proteasome (D). N = 3, * P<0.05, ** P<0.01; *** P<0.001.

Protein degradation is an extremely important component of the PN that eliminates unfolded or denatured proteins, controlling cellular homeostasis [44, 45]. For example, if proper folding does not occur, misfolded proteins are polyubiquitinated and degraded by the 26S proteasome [46]. After observing increased basal levels of UPR and impaired heat shock response in DS cells, we next investigated the ability of control and DS fibroblasts to ubiquitinate and degrade proteins. Immunocytochemical analyses of vehicle (DMSO) and MG132-treated (5μM) fibroblasts showed that immunostaining for ubiquitin was greater in vehicle treated DS vs CTL cells (Fig 8A) and quantification of these images showed a significantly greater abundance of ubiquitinated proteins in these DS cells (Fig 8B). MG132 treatment greatly increased ubiquitin staining in both DS and CTL cell lines; however, 24 hours of accumulation did not yield a statistically significant difference when comparing CTL to DS. We next evaluated a time course of MG132-mediated accumulation of polyubiquitinated proteins in both DS and euploid control fibroblasts (Fig 8C). These data show that DS cells not only possess a higher abundance of polyubiquitinated proteins, but inhibition of the proteasome by MG132 resulted in greater accumulation of these proteins in the DS cells, and at earlier time points, when compared to CTL cells (S2 Fig). Due to these observations of increased protein ubiquitination/accumulation in the DS cells, we next investigated the proteolytic function of the proteasome. Fig 8D clearly shows that DS fibroblasts possess significantly lower chymotrypsin-like and trypsin-like proteolytic activity compared to CTLs. Additionally, we did not observe any DS-mediated alteration in caspase-like activity of the proteasome. These observations indicate that DS cells have errors in protein folding that should promote protein degradation; however, the proteasome of DS cells is unable to effectively degrade these misfolded proteins. Together, these data indicate a disruption of the PN in DS and a tangible mechanism for some of the cellular phenotypes observed in this syndrome.

### DS cells display enhanced loss of viability when treated with ER stressors

**Fig 9:**
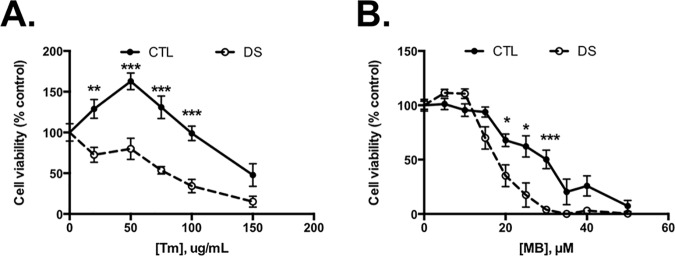
Cell viability in DS cells is altered to a greater extent due to exposure to ER stressors compared to CTL. Cells were treated for 24h with Tm (A) or MB (B) and cell viability was assessed using a WST-1 assay. N = 3–4, * P<0.05; ** P<0.01; *** P<0.001.

The PN is dynamic and must work to strike a balance between protein production and protein turnover, clearing misfolded and aggregated proteins, and must be stress responsive. Decline or disruption of this network is associated with aging and diseases of aberrant protein folding and aggregation [44] with the prediction that a decline in the capacity of the PN can have far-reaching and toxic consequences [45]. Due to these observations, we next compared the impact of compounds that affect the PN on cell viability in DS and euploid control fibroblasts. Fig 9 clearly illustrates that DS cells are more sensitive to the toxic effects of the ER stressor compounds tunicamycin (glycosylation inhibitor) (Fig 9A) and maneb (proteasomal inhibitor [47], thiol-modifying fungicide [48]) (Fig 9B). These data show that impairment of the PN in DS cells renders them vulnerable to cell death when the PN is further challenged, contributing in turn to cell loss and various comorbidities associated with DS.

## Discussion

DS is a genetic condition caused by the presence of three copies of chromosome 21. This genomic imbalance renders the DS population vulnerable to developing several comorbidities at a higher percentage than non-DS population. An epidemiological study concluded that the incidence risk ratio (IRR) for new onset of eye disorders, hypothyroidism and diabetes, of DS individuals compared to CTL, was increased (3.1, 13.1 and 1.3 respectively) during childhood in DS. Also, in DS adulthood, the IRR for epilepsy and intellectual disability is increased to an even greater level compared to other comorbidities (15.2 and 158 respectively)[49]. Previous reports in the literature describe a dysfunction of PN components and modulators as being associated with the above comorbidities. Considering the fact that aneuploidy causes alterations to the proteome [50], this inspired our interest in investigating the protein quality control system in DS cell models.

Although a significant amount of research regarding gene expression in DS models exists [51], information about the maintenance of the proteome in DS is scarce. Reports regarding protein expression in DS [50, 52, 53] provide valuable, but limited, insight into protein quantification and oxidative posttranslational modifications. An important conclusion from these reports is that the increased gene expression is not always accompanied by an increase in protein level, and this conclusion highlights greater need for future DS proteomic investigations. For example, we have presented gene expression data indicating activation of multiple arms of the UPR; however, further investigation into protein abundance and phosphorylation status did not yield similar results. Interestingly, Dephoure and colleagues (2014), using yeast models of aneuploidy and quantitative proteomic techniques, demonstrated a disconnect between observed mRNA level and protein abundance. Specifically, these researchers showed that, in aneuploidy, there is a large, statistically significant number of proteins whose abundance did not exactly correlate with the observed mRNA level [50], which is in line with the data presented here.

A major modulator of the PN that is also involved in the pathology of several comorbidities that appear in the DS is UPR induction. Although there is significant literature describing the role of UPR in AD [54], reports that investigate this significant cell response in DS are lacking. Our data show a modest, yet significant increase in the gene expression of a majority of UPR-related genes. Their transcriptional increase indicates the presence of dysfunctional proteins due to misfolding in DS lymphocytes and fibroblasts, and is also a marker of the cell’s attempt to “fight back” in this scenario as shown in previous research [55, 56]. It should be noted that this gene expression data implies that multiple arms of the UPR are activated. For example, expression of CHOP was approximately 2.5-fold greater in DS cells compared to euploid controls, potentially indicating activation of the PERK-ATF4 arm of UPR. However, CHOP has been referred to as a “cross-roads for multiple signal transduction pathways” [57], and this gene has been reported to be a target of many transcription factors other than ATF4 like AP-1, ETS-1, p53, and ATF6 [57–59]. Lastly, alterations in expression of genes involved in UPR were recently shown to be dysregulated by chronic stress by the Rutkowski laboratory [40].

Although not all of the UPR-related genes were increased in the DS cell models at the protein level, the elevated abundance of XBP1s and ATF6 activation support the existence of a chronic UPR in our cell models of DS. ATF6 is a critical regulator of ER quality control in higher eukaryotes, and its activation has been reported to be an adaptive measure that is essential for the cell to respond to chronic stress [60, 61]. Additionally, up-regulation of XBP1s protein abundance and/or activation of ATF6 in the absence of other UPR markers have been observed by other investigators [62–65], validating our results. The role of XBP1s in dementia is of high significance as it has been shown to exert a protective effect by regulating brain-derived neurotrophic factor (BDNF), which is involved in memory processes [66]. XBP1s has also been shown to regulate the expression of ADAM10, an α-secretase that cleaves β-amyloid and generates non-toxic species [67]. These reports suggest that XBP1s overexpression observed in DS models might be an attempt of DS cells to either protect themselves against toxic β-amyloid species produced as a result of APP overexpression [68], or as a protection against the lack of neurotrophic factors, also observed in DS models [69, 70]. A similar induction of UPR has also been observed in cataracts [71], epilepsy [72], hypothyroidism [73] and diabetes [74]. Lastly, the presence of an UPR and the appearance of these comorbidities in the DS population raises the question of whether there is a link between enhanced levels of UPR in DS and the increased risk of developing these comorbidities in DS individuals.

Work in yeast models has shown that cells carrying an extra chromosome exhibit dysfunctional protein quality control leading to proteotoxic stress [75]. Authors have proven that genes from an extra inserted chromosome produce both increased transcripts and proteins, and attribute proteotoxic stress to this aberrant transcription and/or protein production. Although different models, increased transcription of genes, in response to the presence of increased Hsa21 genes, have been previously shown in DS [25]. Although chromosome number was not directly measured, the cells used in this work having originated from DS individuals, serve as a potential link between aberrant protein production and alterations in chromosomal numbers, being the prime candidate mechanism of the PN dysfunction we have observed. This scenario may also explain the hypothesis that elevated APP gene expression leads to increased β-amyloid production and a link between the early-onset AD pathology developing in DS individuals. The work of Perluigi and colleagues further support [76] a tight relationship between protein oxidation and dysfunctional protein degradation systems. Our work, a report of PN dysfunction in DS, can be viewed as a natural progression of this relationship, since oxidative stress can produce UPR and vice versa [77, 78].

The 26S proteasome is, along with autophagy, the main protein degradation pathway of a cell [79]. While the autophagic pathway was not investigated in this report, previous research has proposed altered autophagic processes in DS [80]. After the induction of UPR, misfolded proteins are retrotranslocated to the cytosol where they are tagged with ubiquitin and guided to the proteasome for enzymatic degradation. [81]. The presence of a two-fold increase in ubiquitinated protein abundance implies the presence of increased misfolded proteins in DS cells and an increased attempt to degrade these proteins. This observation may also be explained by the increased UPR activation through XBP1s and ATF6, since these two transcription factors activate protein degradation [82]. Concerning the impairment of proteasomal activity in DS cells, several scenarios may take place. For example, aberrant production of misfolded proteins is a possible suspect for this event. If the volume of misfolded proteins is higher than the amount the proteasome can handle, then the degradation machinery can be impaired and decreased degradation is observed [83]. Oxidative stress that causes mitochondrial dysfunction and a decline in ATP generation in DS has been previously reported [84]. ATP is the fuel of the 20S component of the 26S proteasome (catalytic compartment that performs the degradation) and decreased ATP observed in DS might provide a rationale for the decreased activity [85]. Additionally, the presence of oxidative stress can directly affect the activity of the 26S proteasome [86, 87]. Oxidative stress also plays a significant role in aging and senescence, and enhanced ROS production has been observed in our model and other DS models [88]. Aging can cause a decrease in protein degradation and the proteasome is a confirmed target of this process [89]. Furthermore, a two-hit scenario can be proposed here: the increased load of misfolded proteins and the decreased activity of the proteasomal proteolytic enzymes describes a system that can no longer cope with proteotoxic stress implying that dysfunction in the PN as a player in the development of the comorbidities observed in DS.

Literature regarding chaperone expression in the DS population is limited. A report from the Lubec research group has shown a decrease of HSP70 RY, HSC71 and GRP75 in the temporal cortex, and an increase of HSP70.1 and GRP78 in the cerebellum of DS patients [90]. However, there is no evidence of chaperome alteration in cell models of DS. Here we report a significant decrease of basal Hsp27 abundance, as well as a non-statistically significant increase of Hsp70 in DS fibroblasts. Members of Hsp27 family are referred to as small heat shock proteins, and several of their functions have been reported, such as inhibition of apoptosis [91] and the acceleration of ubiquitinated protein degradation [92]. The significant decrease in the basal level of Hsp27 may be a contributing factor in the loss of cell viability observed in DS cells. Decreased proteasomal activity in DS has been previously reported [93], with impaired Hsp induction and expression possibly contributing to this observation; however, further investigation is required to confirm this relationship. Regarding AD, Hsp27 has been found to bind to phosphorylated Tau and promote its degradation through the proteasomal pathway, aiding in cell survival [94]. Since phosphorylated Tau is an AD hallmark, decreased abundance of Hsp27 might allow for the accumulation of phosphorylated tau and its aggregation. Hsp70 has received the most attention among chaperone families, as it is a major player in cellular physiology and is implicated in diseases resulting from protein aggregation [95]. From the perspective of the PN, the increase of Hsp70 might serve as a marker of the presence of misfolded proteins and may contribute to the observed activation of the UPR.

The failure of a proper heat shock response in DS fibroblasts is indicative of an inability to cope with increased proteomic stress highlighting the importance of future research in this area. Previous literature on trisomic and tetrasomic cells has shown a similar failure of the PN in response to intense heat stress, as cells could not adequately induce HSF-1, ultimately leading to a disruption in Hsp90 function [96]. Hsp90 is involved in several cellular processes including protein trafficking, protein stabilization and heat shock response [97, 98] and has been shown to interact with Hsp70, together playing a major role in protein folding processes [99]. In resting conditions, HSF-1 is bound by Hsp90 and transcriptional activity is repressed [100]. In stress conditions (e.g. oxidative stress, heat stress) HSF-1 dissociates from Hsp90, trimerizes, and accumulates in the nucleus, where it induces the expression of several genes intended to protect the cell against stress. Hsp genes have an important place in this group of genes as Hsp90, Hsp70 and small Hsps are induced significantly after HSF-1 activation [101]. A defective HSF-1 function in cells of DS origin is therefore a candidate mechanism for the reduced induction of Hsp90 and Hsp70 observed. Adding the failure to modulate the heat shock response highlights the level of dysfunction that the PN faces. An inability to mount a proper response might indeed be the mechanistic basis responsible for the development of comorbidities related to disrupted PN, and detailed mechanistic studies investigating this HSF-1 response in DS are ongoing.

From our results and existing literature, it is obvious that proteotoxic stress is present in the DS cells, as well as a dysfunctional PN in DS models. From the presence of UPR to the increased bulk of polyubiquitinated proteins and from decreased proteasomal activity to the absence of a robust heat shock response, DS cells exhibit an inability to cope with protein misfolding. This manuscript represents a characterization of the stress-responsive modulators of the PN, as well as a major associated degradation pathway. The PN consists of over 1,400 different components, i.e. ribosome, chaperones and co-chaperones, and degradation machinery [21]; therefore, a detailed mechanistic dissection of each individual process is not practical without this basic characterization. Future investigations into the distinct mechanisms involved in ATF6-XBP1s signaling, heat shock response and HSF-1, proteasomal dysfunction, and other PN functions, like autophagy, are logical extensions of this work. As a result of these and future findings, we propose that an ambitious approach to helping DS individuals overcome the comorbidities that affect them, could be, compounds that mitigate the observed disruption of proteome homeostasis, enhance proper folding or induce protein degradation.

## Supporting information

S1 Fig Down syndrome cells are able to induce the ER stress response. DS and CTL LCLs were treated with MB (2.5uM) for 1-24h and Grp78 abundance (A) and eIF2α phosphorylation (B) were measured via Western blotting. These data show that ER stress signaling is not negatively impacted in DS cells.
(DOCX)

S2 Fig DS cells possess greater amounts of ubiquitinated proteins at every time point after proteasome inhibition. CTL and DS fibroblasts were treated with MG132 (10μM) for 0, 1, 2, 4, 8, and 24h, then protein ubiquitination was evaluated using Western blot. These data show that, while variable, the mean amount of ubiquitinated protein was greater in the DS cells compared to controls. N = 3.
(DOCX)

S3 Fig Full blot images for Western blot presented in Fig 3. (DOCX)

S4 Fig Full blot images for Western blot presented in Fig 4. (DOCX)

S5 Fig Full blot images of Western blots of cytoplasmic and nuclear fractions presented in Fig 4. (DOCX)

S6 Fig Whole blot images of Western blots from WT, DP16 and DP17 mice presented in Fig 4. (DOCX)

S7 Fig Full blot images of Western blots presented in Fig 5. (DOCX)

S8 Fig Full blot image of IRE1a inhibitor study (4u8C) presented in Fig 5. (DOCX)

S9 Fig Full blot images of ATF6 Western blots presented in Fig 6. (DOCX)

S10 Fig Full blot images of cytoplasmic and nuclear ATF6 localization presented in Fig 6. (DOCX)

S11 Fig Full blot images of Hsp proteins from 40°C heat stress study presented in Fig 7. (DOCX)

S12 Fig Full blot images of MG132 time course experiment presented in Fig 8. (DOCX)
