# Supplementary material for: The burden of trisomy 21 disrupts the proteostasis network in Down syndrome
Source: PLoS One. 2017 Apr 21;12(4):e0176307. doi: 10.1371/journal.pone.0176307 (PMC5400264; doi:10.1371/journal.pone.0176307)
Supplement: S1 Fig — DS and CTL LCLs were treated with MB (2.5uM) for 1-24h and Grp78 abundance (A) and eIF2α phosphorylation (B) were measured via Western blotting. These data show that ER stress signaling is not negatively impacted in DS cells. (DOCX) [file pone.0176307.s001.docx]

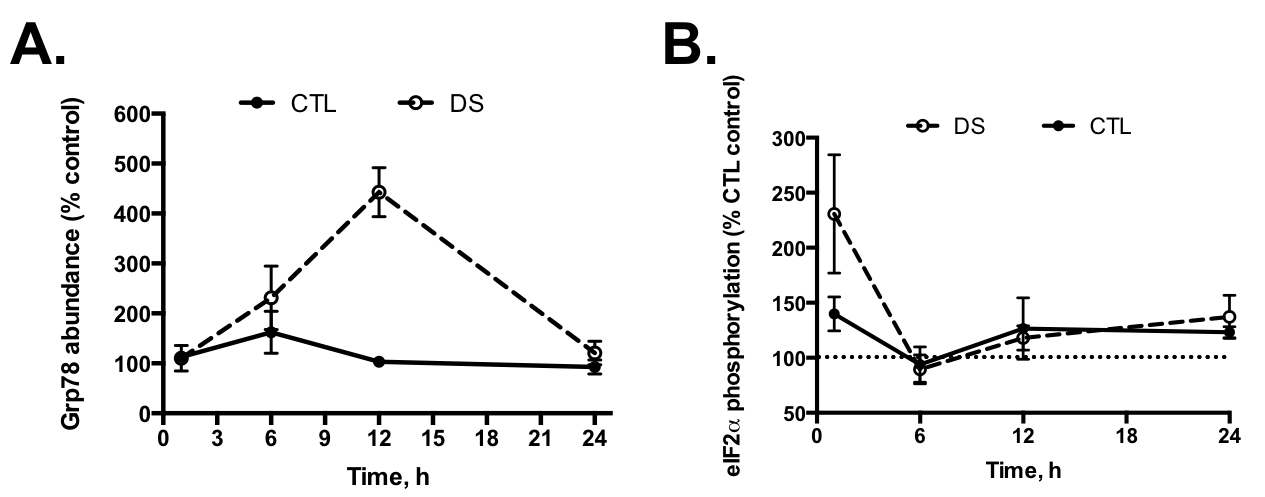


**S1 Fig.** **Down syndrome cells are able to induce the ER stress response.** DS and CTL LCLs were treated with MB (2.5uM) for 1-24h and Grp78 abundance (A) and eIF2α phosphorylation (B) were measured via Western blotting. These data show that ER stress signaling is not negatively impacted in DS cells.
