# Supplementary material for: The burden of trisomy 21 disrupts the proteostasis network in Down syndrome
Source: PLoS One. 2017 Apr 21;12(4):e0176307. doi: 10.1371/journal.pone.0176307 (PMC5400264; doi:10.1371/journal.pone.0176307)
Supplement: S2 Fig — CTL and DS fibroblasts were treated with MG132 (10μM) for 0, 1, 2, 4, 8, and 24h, then protein ubiquitination was evaluated using Western blot. These data show that, while variable, the mean amount of ubiquitinated protein was greater in the DS cells compared to controls. N = 3. (DOCX) [file pone.0176307.s002.docx]

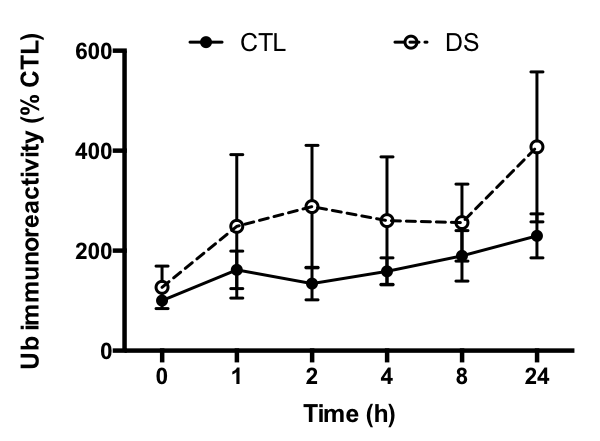


**S2 Fig.** **DS cells possess greater amounts of ubiquitinated proteins at every time point after proteasome inhibition.** CTL and DS fibroblasts were treated with MG132 (10μM) for 0, 1, 2, 4, 8, and 24h, then protein ubiquitination was evaluated using Western blot. These data show that, while variable, the mean amount of ubiquitinated protein was greater in the DS cells compared to controls. N=3.
