# Supplementary figures and images for: The burden of trisomy 21 disrupts the proteostasis network in Down syndrome
Source: PLoS One. 2017 Apr 21;12(4):e0176307. doi: 10.1371/journal.pone.0176307 (PMC5400264; doi:10.1371/journal.pone.0176307)

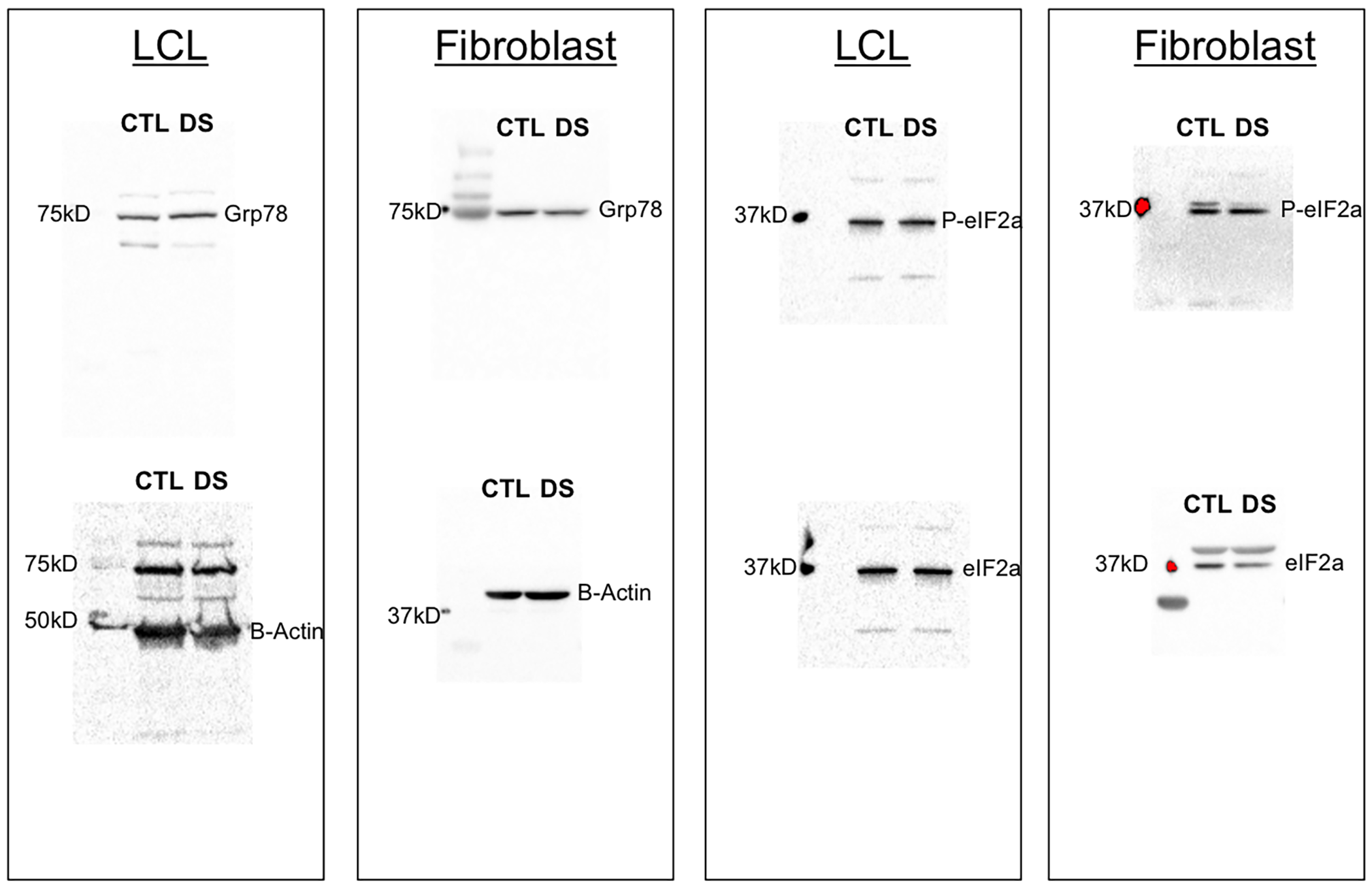


**S3 Fig.** **Full blot images for Western blot presented in Fig 3.**

Supplement: S3 Fig — (DOCX) [file pone.0176307.s003.docx]

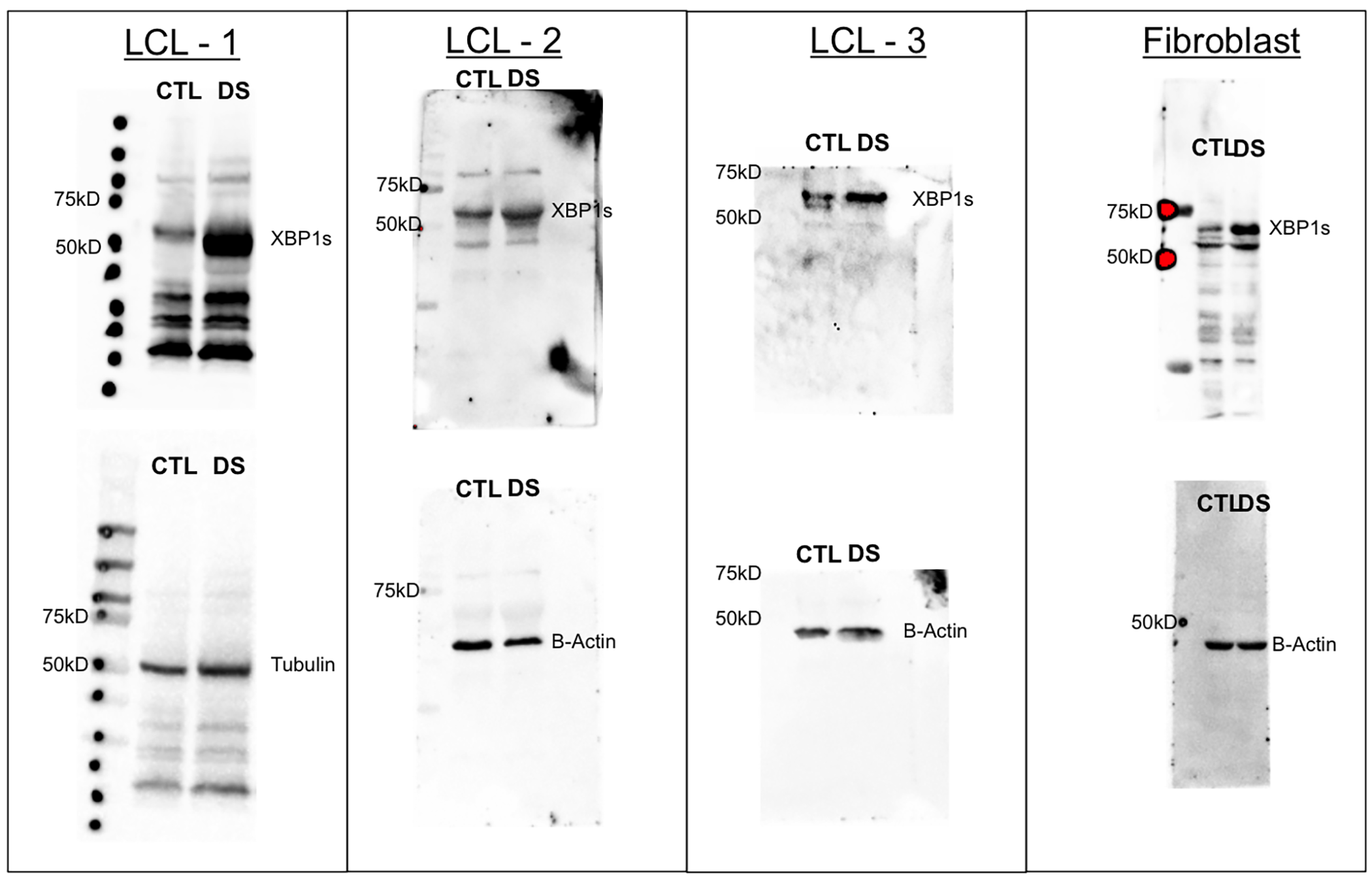


**S4 Fig.** **Full blot images for Western blot presented in Fig 4.**

Supplement: S4 Fig — (DOCX) [file pone.0176307.s004.docx]

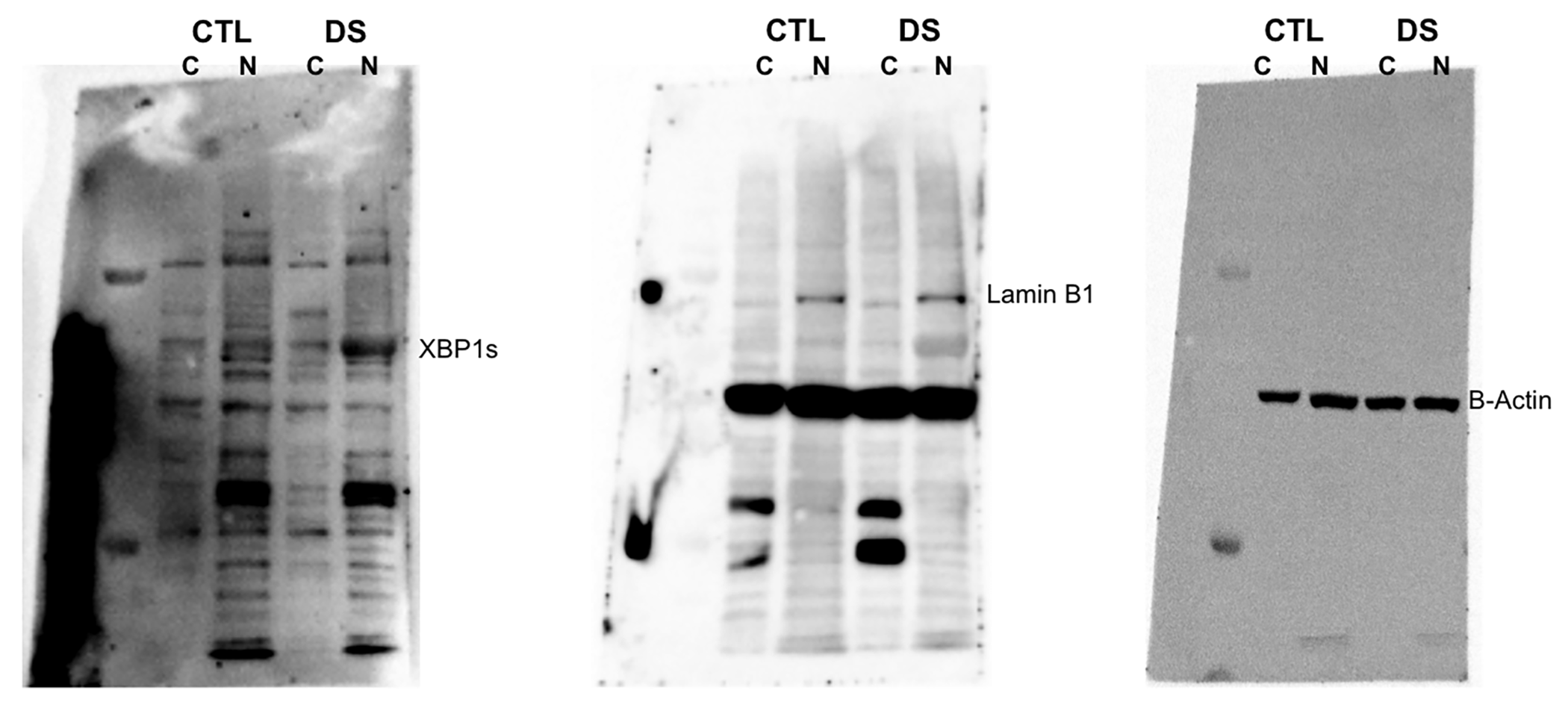


**S5 Fig.** **Full blot images of Western blots of cytoplasmic and nuclear fractions presented in Fig 4.**

Supplement: S5 Fig — (DOCX) [file pone.0176307.s005.docx]

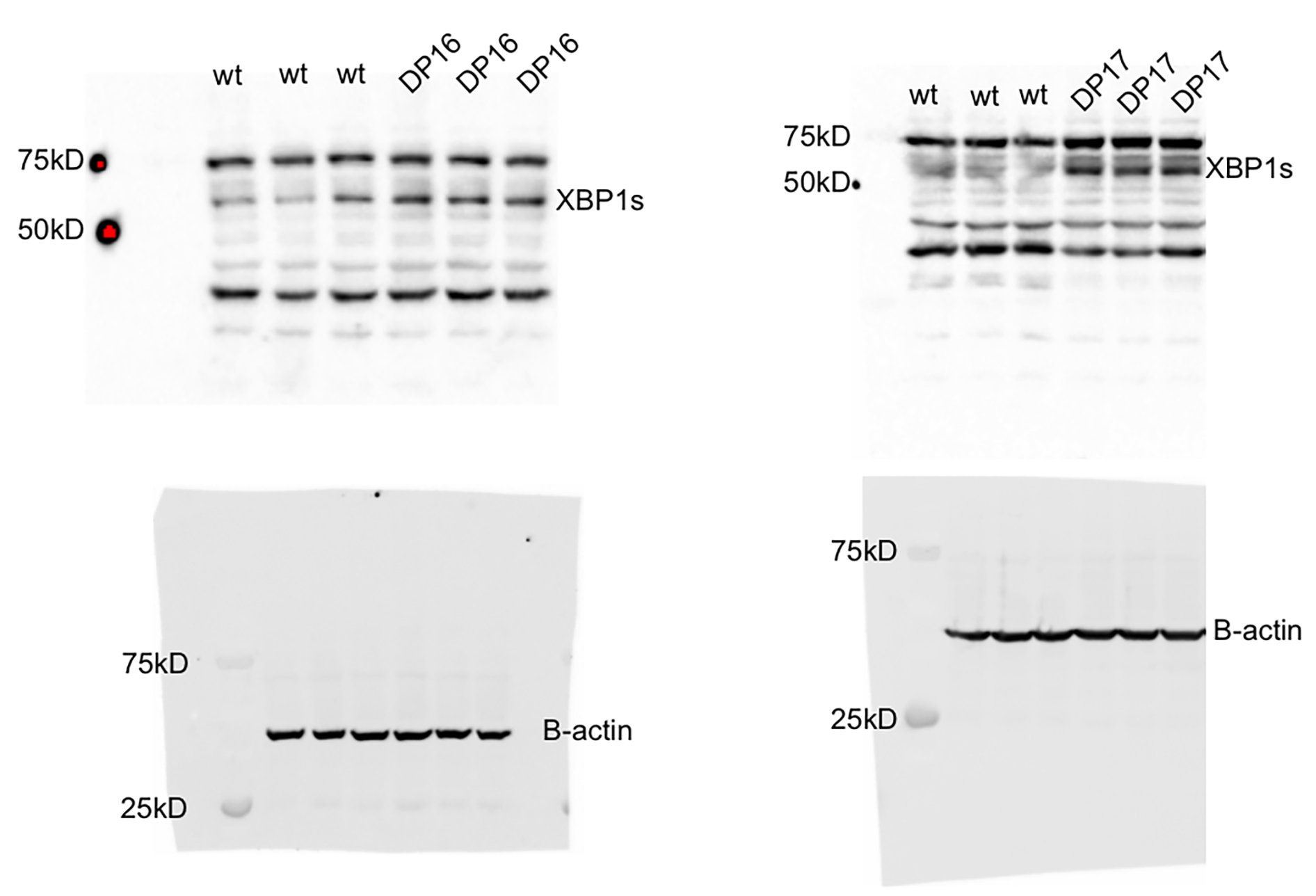


**S6 Fig.** **Whole blot images of Western blots from WT, DP16, and DP17 mice presented in Fig 4.**

Supplement: S6 Fig — (DOCX) [file pone.0176307.s006.docx]

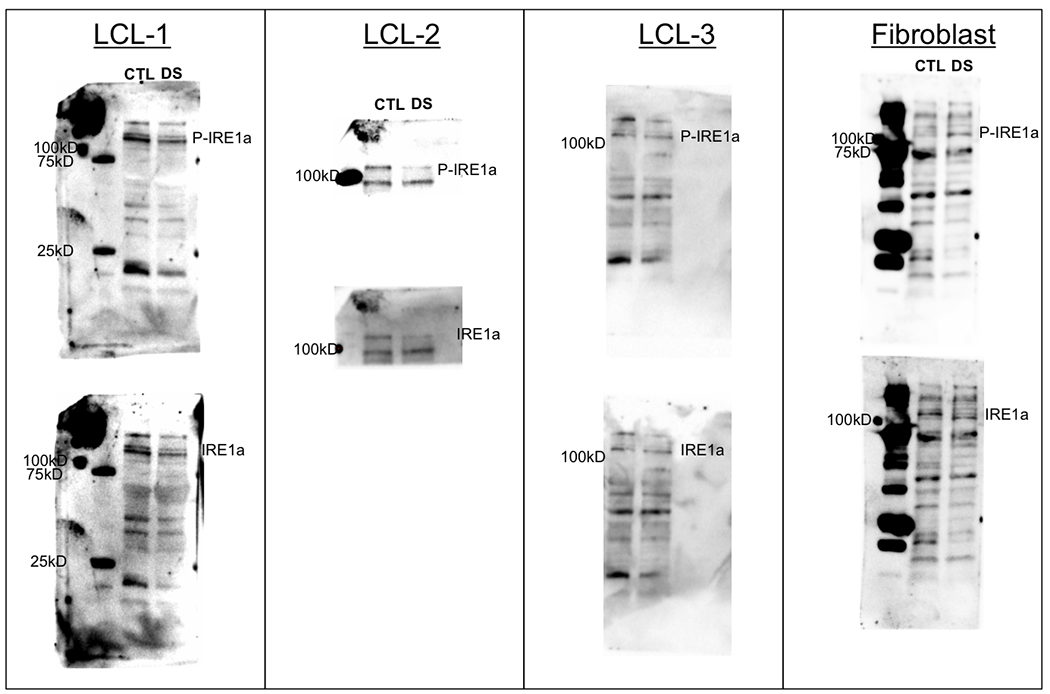


**S7 Fig.** **Full blot images of Western blots presented in Fig 5.**

Supplement: S7 Fig — (DOCX) [file pone.0176307.s007.docx]

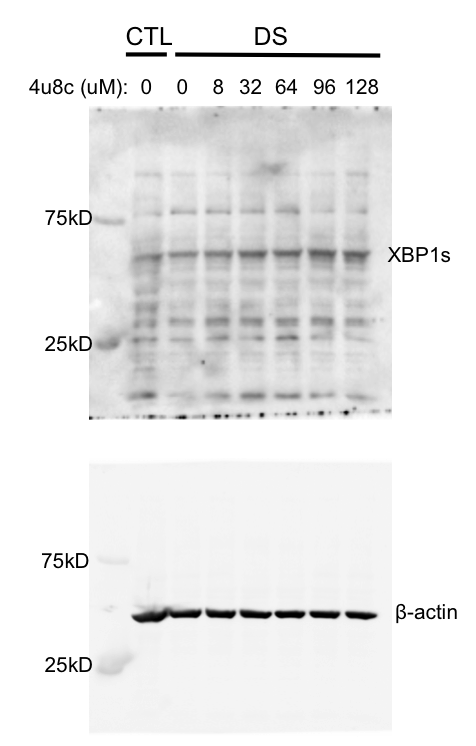


**S8 Fig.** **Full blot image of IRE1a inhibitor study (4u8c) presented in Fig 5.**

Supplement: S8 Fig — (DOCX) [file pone.0176307.s008.docx]

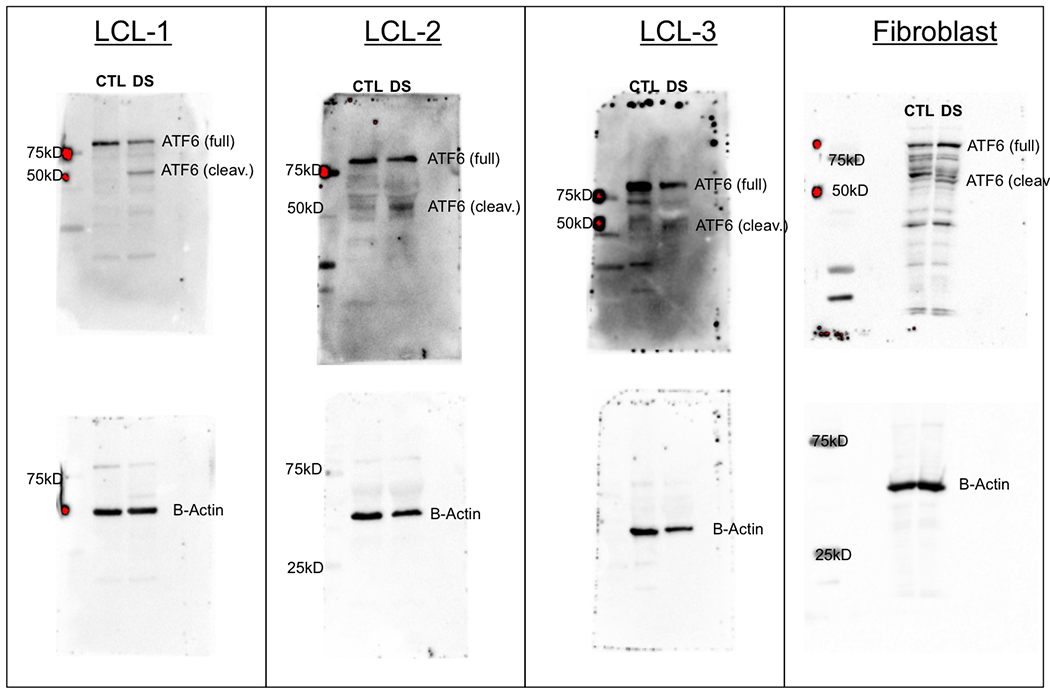


**S9 Fig.** **Full blot images of ATF6 Western blots presented in Fig 6.**

Supplement: S9 Fig — (DOCX) [file pone.0176307.s009.docx]

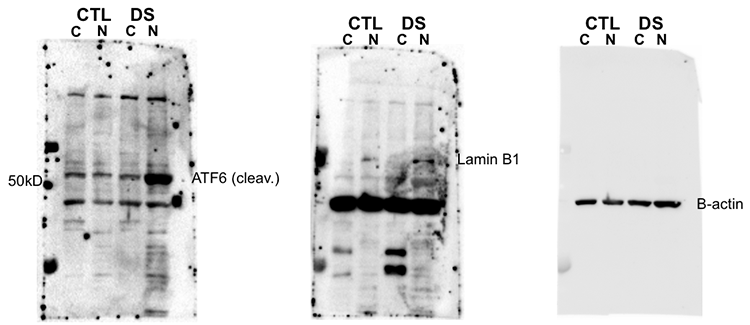


**S10 Fig.** **Full blot images of cytoplasmic and nuclear ATF6 localization presented in Fig 6.**

Supplement: S10 Fig — (DOCX) [file pone.0176307.s010.docx]

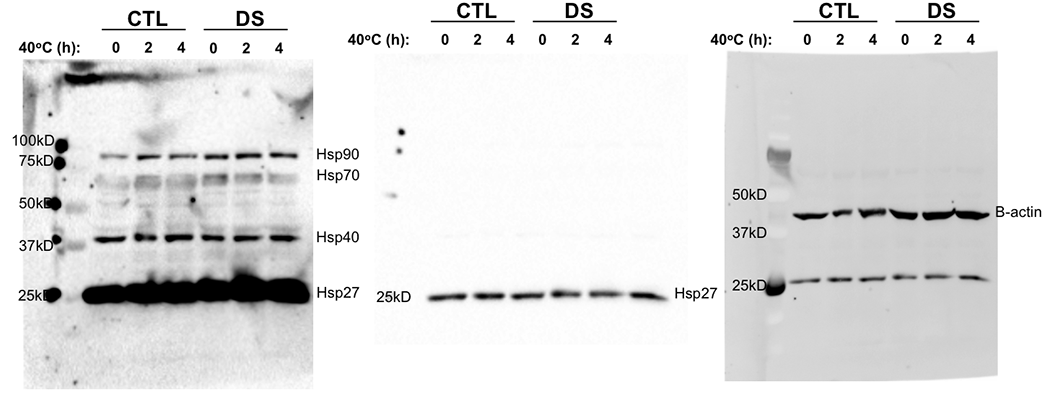


**S11 Fig.** **Full blot images of Hsp proteins from 40oC heat stress study presented in Fig 7.**

Supplement: S11 Fig — (DOCX) [file pone.0176307.s011.docx]

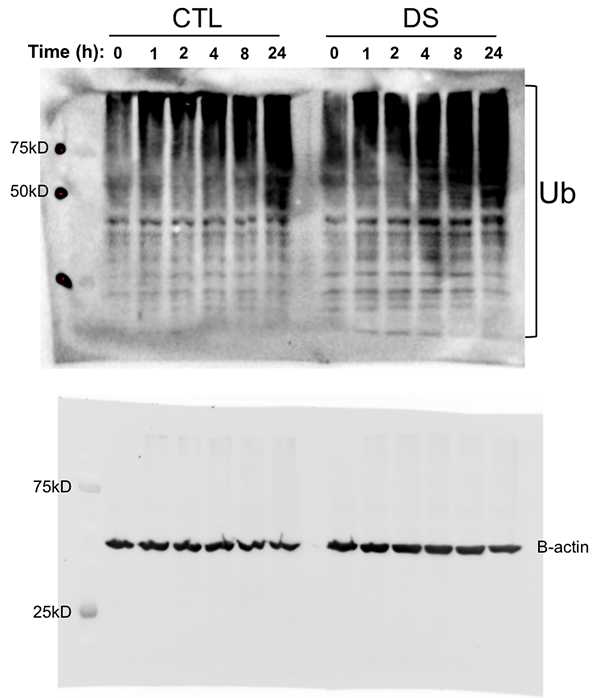


**S12 Fig. Full blot images of MG132 time course experiment presented in Figure 8.**

Supplement: S12 Fig — (DOCX) [file pone.0176307.s012.docx]
